# A convolutional attention mapping deep neural network for classification and localization of cardiomegaly on chest X-rays

**DOI:** 10.1038/s41598-023-32611-7

**Published:** 2023-04-17

**Authors:** Mohammed Innat, Md. Faruque Hossain, Kevin Mader, Abbas Z. Kouzani

**Affiliations:** 1 Department of Electronics and Communication Engineering, Khulna University of Engineering and Technology, Khulna, 9203 Bangladesh; 2 Institute for Biomedical Engineering, Swiss Federal Institute of Technology and University of Zurich, Zurich, Switzerland; 3 School of Engineering, Deakin University, Waurn Ponds, Victoria 3216 Australia

**Keywords:** Engineering, Biomedical engineering

## Abstract

Building a reliable and precise model for disease classification and identifying abnormal sites can provide physicians assistance in their decision-making process. Deep learning based image analysis is a promising technique for enriching the decision making process, and accordingly strengthening patient care. This work presents a convolutional attention mapping deep learning model, Cardio-XAttentionNet, to classify and localize cardiomegaly effectively. We revisit the global average pooling (GAP) system and add a weighting term to develop a light and effective Attention Mapping Mechanism (AMM). The model enables the classification of cardiomegaly from chest X-rays through image-level classification and pixel-level localization only from image-level labels. We leverage some of the advanced ConvNet architectures as a backbone-model of the proposed attention mapping network to build Cardio-XAttentionNet. The proposed model is trained on ChestX-Ray14, which is a publicly accessible chest X-ray dataset. The best single model achieves an overall precision, recall, F-1 measure and area under curve (AUC) scores of 0.87, 0.85, 0.86 and 0.89, respectively, for the classification of the cardiomegaly. The results also demonstrate that the Cardio-XAttentionNet model well captures the cardiomegaly class information at image-level as well as localization at pixel-level on chest x-rays. A comparative analysis between the proposed AMM and existing GAP based models shows that the proposed model achieves a state-of-the-art performance on this dataset for cardiomegaly detection using a single model.

## Introduction

Cardiomegaly is a sign of a cardiovascular disease that abnormally enlarges the heart. It indicates cardiac insufficiency which is found in at least 1 out of 500 in the general population^1,2^. In the U.S. alone, each year around 260,000 people die from cardiac insufficiency^3^. Tavora et al. reported a greater risk of sudden heart death for cardiomegaly^4^. Noirin et al. stated that cardiomegaly is prevalent in stillborn children of diabetes mellitus mothers and may lead to the threat of fetal death during pregnancies^5^. As a life-threatening cardiac condition, it is crucial to recognize the sign and symptoms at an early stage^6^.

Medical images provide vital information to doctors for making diagnostic and therapeutic decisions. The decision-making process involves manual interpretation of the images. To further enrich decision-making and thus strengthen patient care, medical image processing field is devoted to understanding and enhancing the process of clinical interpretation. The classical problems in medical image pre-processing such as segmentation, identification of anomalies and personalized diagnosis are benefitting from the deep learning algorithms^7,8^ and big data^9,10^. Also, the latest accessibility of large-scale medical information encourages a more difficult objective towards causal, explainable, and universal visual medical diagnosis. Thus, in a clinical research area, visual explanation supporting the outcomes of classification, such as spatial region or segmentation^11–13^ of abnormality locations, is an inevitable component of clinical diagnosis. It is therefore crucial that the image processing techniques should be able to provide high precision with both classification results and the associated visual explanation. Moreover, the efficiency of a reliable model heavily depends on the training data in a fully supervised environment. A large, labelled image dataset is often needed to accomplish acceptable generalized performance. However, it is often tedious to perform time-consuming annotation of the targets. Therefore, reducing the annotation costs for locating and detecting objects can be important.

Over the last few years of advancement in artificial intelligence, researchers developed deep learning algorithms for medical image analysis^7,14^. Torres-Robles et al.^15^ proposed a neuro-fuzzy classifier for the detection of cardiomegaly in digital chest radiography. The work used classical morphology operations to segment the lungs for a neuro-fuzzy classifier and thus obtained the feature values to measure heart enlargement. Sema Candemir et al.^16^ reported a Transfer Learning approach to classify cardiomegaly disease by observing several ConvNet architectures. Ilovar et al.^17^ performed an analysis of radiograph and detection of cardiomegaly which employed custom image processing method and defined an edge detector to measure the heart’s and chest cavity’s width. Qiwen Que et al.^18^ presented a procedure to detect cardiomegaly by combing the U-Net model for image segmentation with the DenseNet model as a baseline. In this study, Cardiothoracic Ratio (CTR), calculated from U-Net, was used as a diagnostic metric. The work combined the medical criterion and the deep neural nets to detect heart disease that led the architecture to a much complex system. Takayuki Ishida et al.^19^ presented a computerized system to determine CTR based on an edge detection technique and gray-level histogram to analyze with feature analysis. Pranav Rajpurkar et al.^20^ proposed a 121-layer ConvNet model to detect all 14 diseases^21^ in the chest. J. M. Wolterink et al.^22^ used a dilated convolutional layer for the segmentation of the myocardium and blood pool in cardiovascular MR with congenital heart disease. Yan Shen et al.^23^ applied the routing-by agreement method to classify thoracic diseases including cardiomegaly and used Grad-CAM for the model interpretability.

Classification of the whole-image^24^, the region-based object detection^25^ and the semantic segmentation^26^ have been developed with the advancement of supervised convolutional neural network. Implementing such networks require advanced network engineering as well as a huge amount of precise training pairs. In the case of semantic segmentation or region-based object localization, annotating the precise contours of the target are often tedious to specify and time-consuming as well. Que et al.^18^ presented a system that combined the U-Net and DenseNet model to find the presence of cardiomegaly. It required network designing for both segmentation and classification that brought challenges of accurate image-level labels specification as well as annotation of precise contours of the targets.

Some supervised learning methods^27–29^ showed the promising result of the auto-localization to minimize the annotation effort. However, the region-based procedure for object detection generally uses object proposal pipelines to detect the proper candidate^30,31^ whereas the pixel-level localization procedure^15,21^ try to predict each position on the feature maps. In this work, we examine the GAP system and add a learnable weighting term to design the Attention Mapping Mechanism (AMM) for the salience fields, which show the symptoms of cardiac insufficiency aligning with the visualization and interpretation. At a high-level, an attention mechanism allows a system to concentrate on relevant parts of the input more than the irrelevant parts. The soft attention model-based neural machine translation (NMT)^32^ method has become the state-of-the-art approach, compared with other statistical machine translation (MT) methods, and has been used effectively for computer vision problems^33,34^. It looks for the relevant part of the input to the final prediction. In our experiment, we adopt some of the advanced ConvNet architectures^35–37^ with the proposed Attention Mapping network to build Cardio-XAttentionNet. The AMM explicitly enables the ConvNet to have precise localization capabilities despite being trained on only image-level labels. In contrast to the typical technique of identifying computer vision objects by predicting the bounding boxes, this study tackles both classification and localization concurrently with only image-level labels.

**Figure 1:**
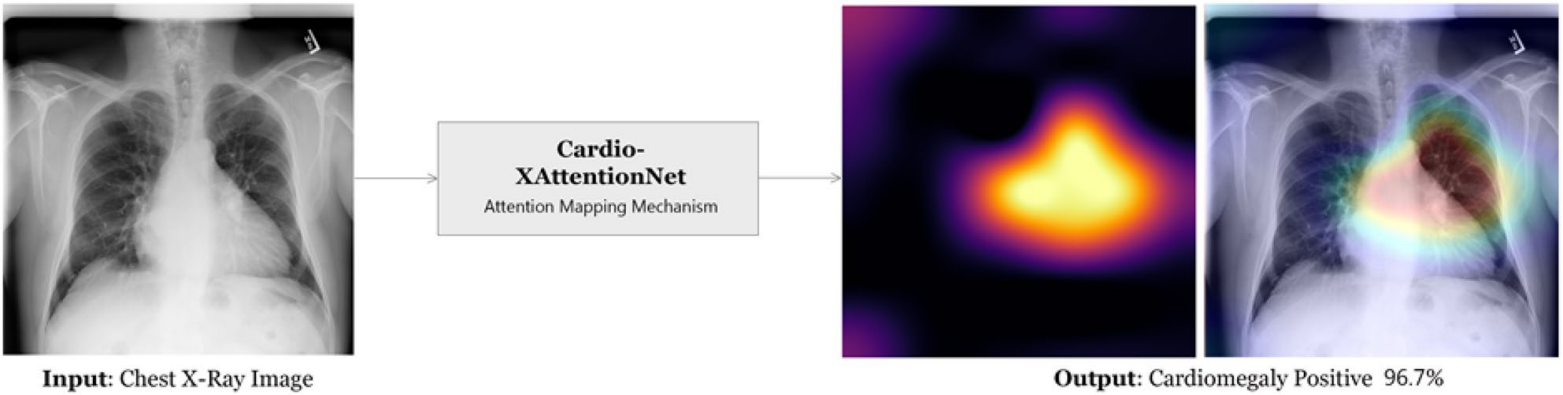
Cardio-XAttentionNet (CXA_Dense121) model, based on DenseNet-121 with Attention Mapping Mechanism, generating accurately positive outcomes with the most indicative region mapping for cardiomegaly, addressing class information and localization on the frontal-view chest X-Ray image. On left side, the original image. On right side, the attention map, and the superimposed outcomes with intensity factor 0.2.

As shown in Fig. 1, our model Cardio-XAttentionNet takes frontal-view X-ray image of chest resulting in the probability of cardiomegaly along with an attention heat-map at pixel-level to address localization. Cardio-XAttentionNet is trained with only image-level labels of the recently published ChestX-Ray14 dataset^21^. To evaluate the empirical evidence of the proposed attention mapping network, we experiment on some of the advanced ConvNet architectures. In our experiment, we use Densely Connected ConvNet^35^, Deep Residual Learning^36^, and Inception-ResNet-v2^37^ as a baseline model which improves the flow of feature information and gradients through the whole network. We reinforce their capability by incorporating with the attention mapping network build top of each model for end-to-end training from scratch. We examined each of these models and came up with the network that had the higher precision.

Specifically, the contributions of this work are:

- A soft Attention Mapping Mechanism (AMM) that enhances the global average pooling (GAP) method by providing a weighting parameter with a range of 0 to 1.
- The Cardio-XAttentionNet, a complete machine learning model that has been built with AMM to perform image-level classification and pixel-level localization tasks concurrently, using only image-level labels as input. Through its training, the model has proven to be effective in successfully carrying out these tasks.
- Comparing the proposed AMM (Weighted GAP)-based models and GAP-based models illustrate the efficacy of AMM module in detecting cardiomegaly and present a wider scope of adaptability.

## Materials and methods

In this section, we describe the ChestX-Ray dataset that is used in this work and demonstrate the model training setup and evaluation methods. This section also describes the data pre-processing steps and finally it discusses about the proposed methods.

### Data set and model settings

#### ChestX-ray data set

The National Institute of Health (NIH) Clinical Centre, a research hospital of USA, recently published over 100,000 anonymized chest x-ray frontal-view images^21^. NIH collected the scanning dataset of over 30,805 patients including many with sophisticated lung diseases. All images are in high resolution (1024 × 1024). Each image marked with up to 15 distinct thoracic categories, including atelectasis, cardiomegaly, effusion, infiltration, mass, nodule, pneumonia, pneumothorax, consolidation, edema, emphysema, fibrosis, pleural thickening, hernia, and normal images. Although the image label is extracted with natural language processing (NLP), the publisher ensures that the NLP labelling precision exceeds the accuracy of 90%^21^. However, the ChestX-ray14 offers 14 class labels and a limited amount of boundary boxes, as ground-truths for fields of localization concern, making it a classic weakly-supervised learning problem^38^.

#### Problem formulation

The cardiomegaly detection task is a binary classification problem along with pixel-level localization. The input is a frontal-view chest X-ray Image *X* and the output is a binary label *y: {1, 0},* indicating the appearance or absence of cardiomegaly respectively along with producing an attention heat map on the identified zone for the presence of cardiomegaly in X-ray images. For a single example in the training set, we optimize the binary cross-entropy loss, stated in Eq. (1):

1 $$L(X,y)=\left\{\begin{array}{l}-y\mathit{log}C(Y=1|X)\\ -(1-y)\mathit{log}C(Y=0|X)\end{array}\right.$$

where $C (Y=i|X)$ is the class probability that the network assigns to the label *i*.

#### Hyper-parameters and model training

The complete model (Cardio-XAttentionNet) has been trained end-to-end from scratch, initialize the weight with glorot uniform initializer^39^. In such process the pre-trained weights from ImageNet can also be used with transfer learning. However, this work is particularly motivated to use such training from scratch considering the fact that ImageNet weights are mainly constructed using natural images. Furthermore, a successful transfer learning requires a significant resemblance between the images used for training and those used for the target application^40^. The network is trained using Stochastic Gradient Descent (SGD) with momentum of 0.9. We have used the mini batches of size 16 and initialize the learning rate at 0.0001 which anneal generally by a factor of 0.3 after 15 epochs each time the validation loss stops improving and saves the model with the lowest validation loss. The model is developed to take an early stop by monitoring the validation loss at patience 40. We have used Keras^41^ deep learning framework to realize the deep learning algorithms and it is experimented on Windows OS having core i7 7th 32 GB RAM with GeForce GTX 1070.

#### Evaluation method

After developing the model, the test dataset is used to assess the model. The parameters required to obtain the four readings: accuracy, precision, recall, and f1-score are True Positive (TP: the number of cases properly predicted as specified), False Positive (FP: the cases incorrectly predicted as necessary), True Negative (TN: the number of cases properly predicted as not necessary) and False Negative (FN: the number of cases wrongly predicted as not necessary). As calculated, the Accuracy, Precision, Recall, and F1-score are stated in Eqs. (2), (3), (4) and(5) respectively.

2 $$ Accuracy = \frac{TP + TN}{{TP + FP + TN + FN}} $$

3 $$ \Pr ecision = \frac{TP}{{TP + FP}} $$

4 $$ Recall = \frac{TP}{{TP + FN}} $$

5 $$ F1 - Measure = \frac{2*Precision*Recall}{{Precision + Recall}} $$

In relation to the above assessment criteria, we also use the Area Under Receiver Operating Characteristics (AUROC) to assess the merits of the model. The Receiver Operating Characteristics (ROC) curve is developed at different threshold values by plotting the True Positive Rate (TPR) against the False Positive Rate (FPR), as stated in Eqs. (6) and (7) respectively. The stronger the classifier, the greater the region under the ROC curve. If the model operates well, a strong classifier will provide TPR close to unity while maintaining FPR close to zero.

6 $$ TPR = \frac{TP}{{TP + FN}} $$

7 $$ FPR = \frac{FP}{{FP + TN}} $$

As shown in Table 1, we also computed the confusion matrix to demonstrate our best model. It is a table that is used to evaluate the performance of a binary classifier. It compares the predicted classifications with the true classifications to understand the effectiveness of the model.

**Table 1:** Confusion matrix table.

	Predicted label	
True label	TN	FP
	FN	TP

### Data preprocessing

In this study, we have used normal (healthy) images and cardiomegaly conditioned images. The cardiomegaly cases in this dataset are 2776 compared to the non-cardiomegaly (healthy) cases of 60,361. From this dataset^14,21^, the training (80%) and testing (20%) datasets are prepared with a patient-wise official split, which creates the training dataset of 52,207 images (1707 cardiomegaly and 50,500 normal images) and a testing dataset of 10,930 images (1069 cardiomegaly and 9861 normal images). Furthermore, we also create 10% validation set (or development set) from the training set which consists of 5221 images including 171 cardiomegaly and 5050 normal images. This validation dataset is used for hyper-parameter optimization and typically smaller than the size of test dataset. In our experiment, the primary picture is scaled down to 224 × 224 pixels for fast processing. We also conduct min–max normalization to decrease computational costs as the ConvNet converges on [0, 1] information much quicker than [0, 255].

**Figure 2:**
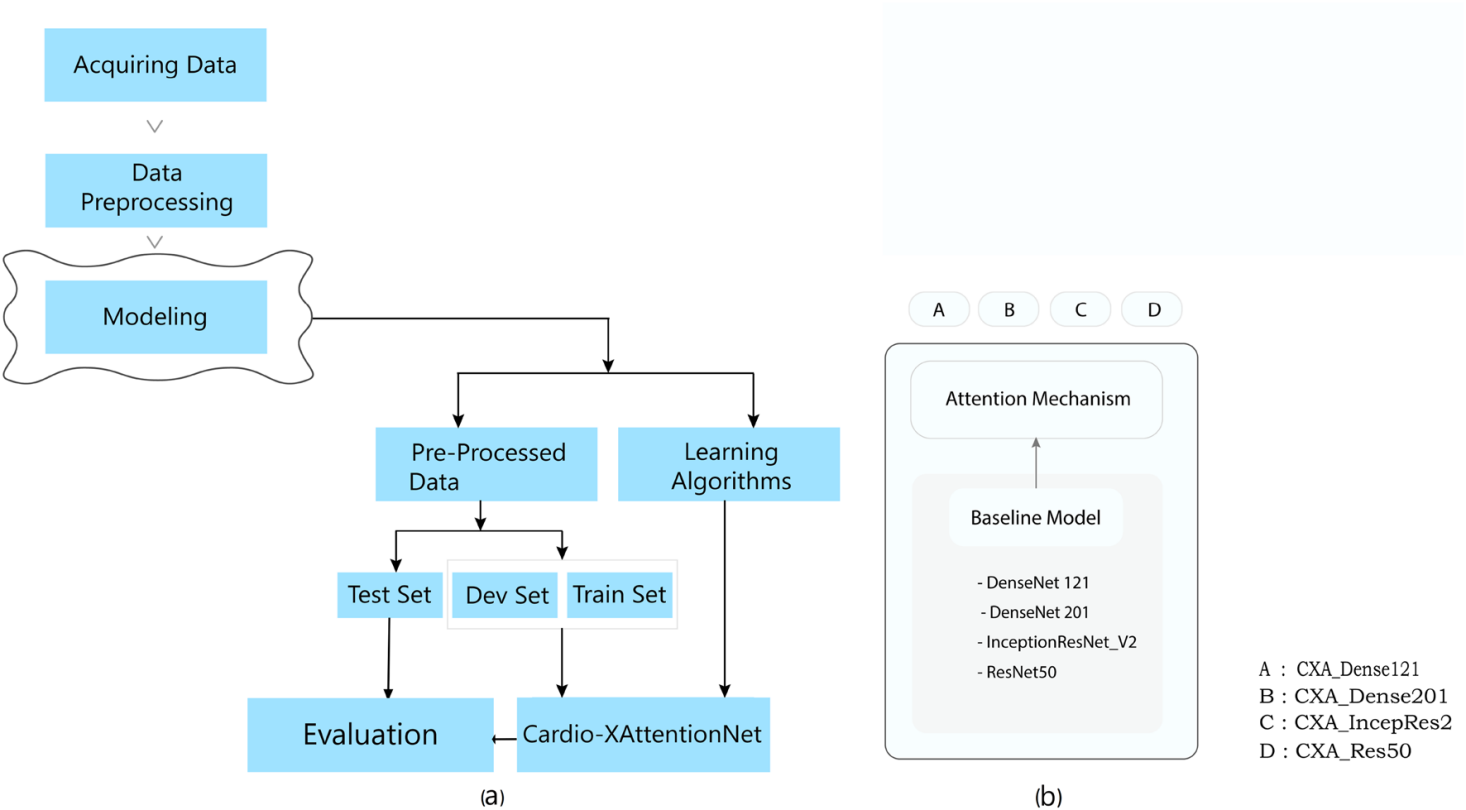
(**a**) Model’s Framework: pre-processing the acquired data to make the training part (train set and dev set) for end-to-end training and learning algorithms to build the models; test set is used to final assessment of the models. (**b**) Architectural design of the learning algorithms. A: CXA_Dense121, B: CXA_Dense201, C: CXA_IncepRes2 and D: CXA_Res50 are built by integrating AMM with the baseline models of DenseNet121, DenseNet201, Inception_ResNet_V2 and ResNet50 respectively.

The framework of the model is shown in Fig. 2a. As we have a significant class imbalance in the datasets between Cardiomegaly and Non-Cardiomegaly cases, we estimate the class weight and use it during the model training. We have taken the training part (training set and development set) for end-to-end training and learning algorithms to build the models. The development set has been used for the optimization of hyperparameter and for choosing the prevailing model. Finally, the test set has been used for the final evaluation of the models.

#### Data augmentation

**Figure 3:**
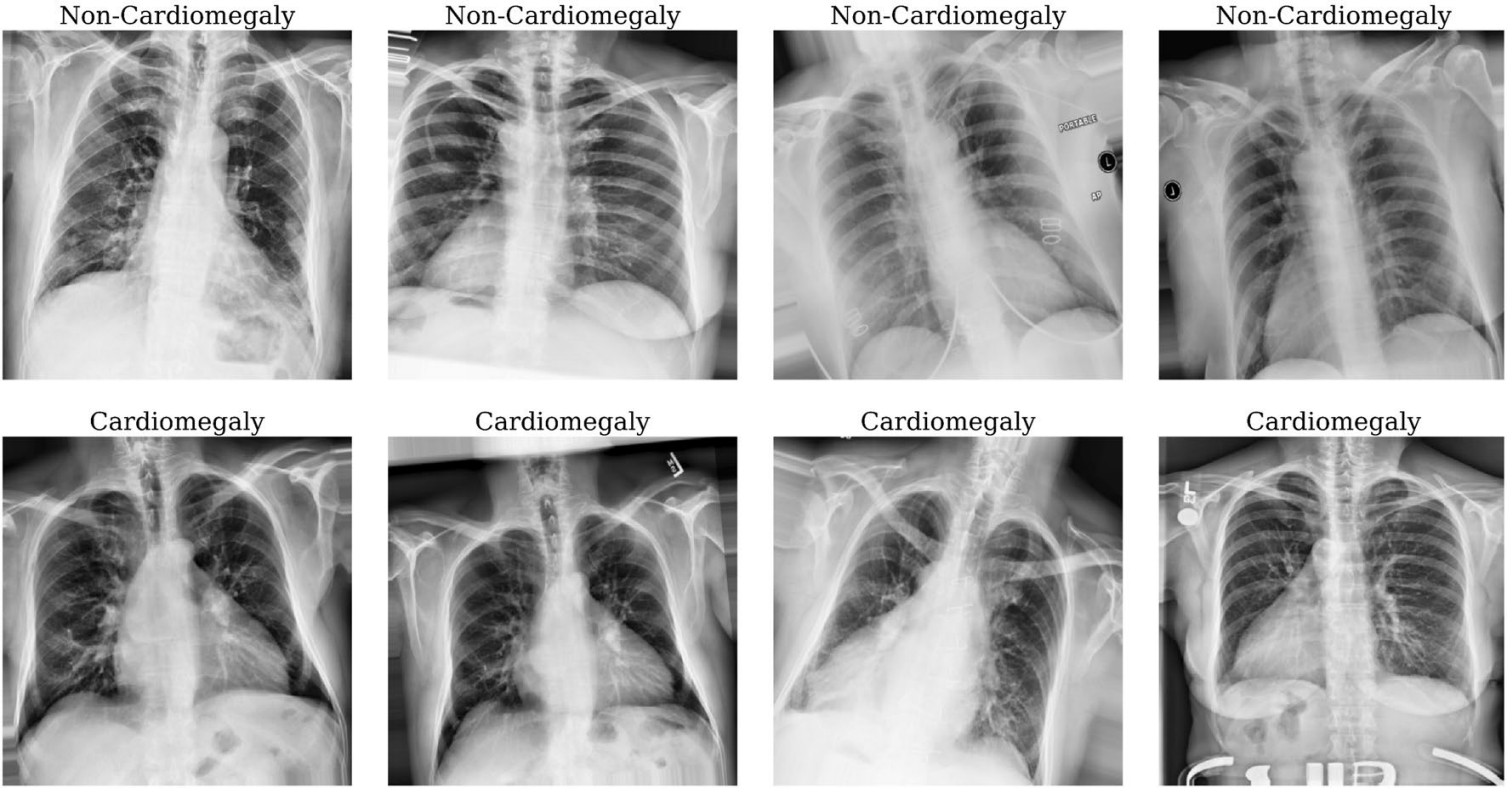
Pre-processed and augmented x-ray images used as training samples. The methods used for augmentation are shifts, rotations, flips, shear, brightness, and zooming. Cardiomegaly and the healthy images were mapped as 1 (Cardiomegaly) and 0 (non-Cardiomegaly) respectively.

In this work, we apply online image data augmentation methods to the training dataset. Online augmentation methods are robust to overfitting and natural variance of objects since the model never sees exactly the same training image twice. Thus, they also have advantage of saving disk space^42^. Figure 3 shows some typical augmented images where the amount of the training data is enhanced arbitrarily by generating an altered image version. The methods used for the augmentation are shifts, rotations, shear, brightness, and zooming. Creating such image variability in the training set will usually enhance the capacity of the fitted model for its generalized applications^43^.

### Cardio-XAttentionNet

Generally, the operation of Global Max Pooling (GMP)^44^ and Global Average Pooling (GAP)^27^ have been considered for precise location of the objects. In GMP, only the maximum value is considered as the final output by discarding other possible relevant details. On the other hand, GAP requires all inputs as the final production. However, it is unable to specify which inputs require more attention to show that some areas are more significant than the others. Since GAP contains all values, we examine the operation of GAP and add a learnable weighting term to develop the AMM. To implement the complete learning algorithm, we remove the final fully connected layer of the baseline model and replace it with the AMM. Next, we add a binary output where we apply sigmoid non-linearity. It is to be noted that the layer of the AMM module consisting this sigmoid nonlinearity ultimately builds the fully connected layer for the classification tasks. Finally, we accomplish that our proposed Cardio-XAttentionNet along with the DenseNet-like base model appears as the most generalized model which produces strong results in addressing cardiomegaly class information and localization concurrently, and substantially outperforms many other models. We demonstrate that such pixel-level attention algorithm trained only on image-level labels can efficiently highlight the areas of salience to demonstrate the symptoms of cardiac insufficiency aligned with visualization and perception.

In our experiment, we have used Densely Connected ConvNet^35^, Deep Residual Network^36^, and Inception-ResNet-v2^37^ which performs as a base model of the Cardio-XAttentionNet. Figure 2b demonstrates the architectural design of the learning algorithms. As shown in figure, the learning algorithms: CXA_Dense121, CXA_Dense201, CXA_IncepRes2 and CXA_Res50 are developed by combining the proposed Attention Mechanism with, DenseNet-121, DenseNet-201, InceptionResNet_V2 and ResNet50 respectively. We experiment with each of these promising systems and finalized it with the most established network. As previously mentioned, we remove the final fully connected layer of the base models and replace it by the Attention Mapping Mechanism (AMM) with a binary output where we apply sigmoid non-linearity. Finally, we achieve that Cardio-XAttentionNet based on DenseNet-121 with AMM, which appears as the most generalized model producing strong results in cardiomegaly classification and localization simultaneously and significantly outperforms the other models.

#### Convolutional neural networks

As previously mentioned, the base models of our experiment are ResNet-50, DenseNet-121, DenseNet-201 and InceptionResNet_V2. These models have dominant performances in ILSVRC competitions^45^. We select these ground-breaking network concepts to build the model with the proposed AMM. Our proposed AMM can also be easily extended to any other advanced ConvNet architectures.

As shown in Table 2, after removing the final classification layer and global pooling layer, an input image with shape (*H*, *W*, *C*) generates a feature tensor with shape (*Fx*, *Fy*, *Fk*). Here *H*, *W* and *C* are the height, width, and channel numbers of the input image respectively, and *Fx*, *Fy*, *Fk* are the height, width, and channel numbers of the feature maps. The output of these networks encodes the image information into an abstract collection of function maps.

**Table 2:** Size of input images (H, W, C) and generated feature tensor (F_x_, F_y_, F_k_) using four different base model.

Base ConvNets	*H*, *W*, *C*	*F*_x_, *F*_y_, *F*_k_
InceptionResNet_V2	224 × 224 × 3	5 × 5 × 1536
ResNet-50	"	7 × 7 × 2048
DenseNet-121	"	7 × 7 × 1024
DenseNet-201	"	7 × 7 × 1920

#### Attention mapping mechanism

As explained before, the different inputs require different level of care. However, the available GMP or GAP operations are limited to such operation since there are no learnable parameters in it. The developed Attention Mapping Mechanism (AMM) is used in this case to generate a learned weight map to create a spatial mask for the feature maps of the base model. To implement the mechanism, we take the feature maps *F ∈ ℝ (Fx, Fy, Fk)* from the last convolution layer of the baseline model. The class and localization information of the target should already emerge in the feature maps, *F* now. Thereafter, we apply the Batch Normalization (BN) to feature maps* F* to further accelerate network training^46^.

**Figure 4:**
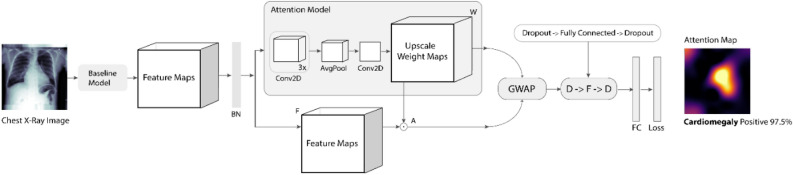
The network architecture of the Attention Mapping Mechanism (AMM) where BN, F, W and A represent Batch Normalization, output feature maps of the base model, learned weight maps of the attention model and weighted feature maps respectively. GWAP represent the Global Weighted Average Pooling operation that perform on weighted feature maps (A) and learned weight maps (W). Layers with concerned filter sizes of the AMM are demonstrated in Table 3.

Figure 4 illustrates the network architecture of attention mapping mechanism (AMM) where an Attention Model is used to obtain a weight *W* that learns a spatial mask for the feature maps *F* to produce region wise attention. The learned weight should be a positive value and cannot be zero either, such as ${\forall W}_{K}\left(x,y\right)\ge 0$ and ${\sum }_{x,y}^{k}W\ne 0$. Generally, the network architecture is like Network-In-Network as demonstrated in^47^. Table 3 gives the detail of each layer of the model. As shown in table, the model largely consists of 1 × 1 convolutional layer with exponential linear units (ELU) activations^48^.

**Table 3:** Layers of the Attention Mapping Mechanism (AMM).

Layers	Kernels	Size, Stride, Pad	Description
Train Data	–	224 × 224	RGB image crop
Test Data	–	Full Size (1024 × 1024)	RGB image full
Base Model	–	–	DenseNet-121 | DenseNet-201 | Inception_ResNet_V2 | ResNet-50
Conv1	128	1 × 1, 1, ‘same’	ELU
Conv2	32	1 × 1, 1, ‘same’	ELU
Conv3	16	1 × 1, 1, ‘same’	ELU
Avg-Pool	–	2 × 2, 1, ‘same’	–
Conv4	1	1 × 1, 1, ‘valid’	Sigmoid
Conv5	(Same as base model)	1 × 1, 1, ‘same’	Linear + Trainable: False (Hard-Coded Conv)
Conv6	–	–	Conv5 (Conv4)
Conv7	–	–	Multiply ([Conv6, Base Model)]
GAP (Conv6 and Conv7)	–	–	Rescale-Gap + Dropout
Dense	–	128 units	ELU + Dropout
Dense	–	1 unit	Sigmoid

Conv refers to the Convolution operation.

During training, we scale down the image into 224 × 224 pixels, where testing is performed on the entire input image.

In the last layer, we apply a sigmoid non-linearity activation function to obtain a weight *W* that will be used to make the features attentive in the feature maps *F*. For each feature, thus, the attention model gives a learned weight from 0 to 1 according to sigmoid non-linearity. Then the output feature dimensions of the learned weight maps of the attention model rescale back to the original number of base model feature *F* using a hard-coded operation where we ignore the bias term and set the network layer non-trainable. Next, we use this attention model to weight the regions of the feature of *F*; since some of the regions are more relevant than others. Thus, the high weighted features will get more attention to the weighted mean than the low weighted features. Before the final output layer, we perform the global weighted average pooling (GWAP) operation on the convolutional weighted feature maps (*A*) of the baseline model with the learned weight maps (*W*) of the attention model as shown in Fig. 4. With this connectivity framework, we can effectively distinguish the relevance of the spatial image regions by re-projecting the weight maps of the output layer onto the convolutional feature maps.

Let *F*_*k*_ be the kth feature map and *W*_*c,k*_ be the learned weight in the final classification layer of the attention model for feature map *k* leading to pathology *c*. We obtain an attention feature maps *A*_*c,k*_ of the most salient features in classifying the image as having pathology to class* c*. *A*_*c,k*_ is the results of the element-wise products (⊙) of feature maps *F* using their associated weight maps *W*, which obtained from the proposed AMM.

8 $$ A_{c,k} (x,y) = F_{k} (x,y) \odot W_{c,k} (x,y) $$

where *1* ≤ *x* ≤ *Fx, 1* ≤ *y* ≤ *Fy*. Here *Fx*, *Fy*, and *Fk* denote height, width, and channels of the feature maps respectively.

The higher the weighting term of *W*_*c,k*_, the higher the values in *A*_*c,k*_ production. Therefore, we use the weighted average to calculate the value of *A*_*c,k*_*(x,y),* so that the scale is independent of the region of attention. To achieve this, we employ the GAP layer which sums the spatial information for both *A*_*c,k*_ and *W*_*c,k*_ as it is more prevalent to a spatial translation of the input. Both feature maps have the spatial mask information and should emerge the localization information of the target along with classification confidence. We can now normalize the weighted feature maps *A*_*c,k*_ by the learned weight maps *W*_*c,k*_ as follows:

9 $$ f_{GWAP} = \frac{{\sum\limits_{c,k} {A(x,y)} }}{{\sum\limits_{c,k} {W(x,y)} }} = \frac{{\sum\limits_{c,k} {F_{k} (x,y) \odot W_{c,k} (x,y)} }}{{\sum\limits_{c,k} {W(x,y)} }} $$

where *f *_*GWAP*_ ∈ ℝ^k x 1^. Next, we grab the output of the attention map and use the Dropout layer^49^ followed by the fully connected layer for the classification. A binary cross-entropy loss function is generally used for training the entire network. After the training, the model produces accurate class labelling along with simultaneously anticipating the relevant areas in a forward pass. By this, along with the classification, the proposed model (Cardio-XAttentionNet) can create an attention map at pixel-level to address localization for cardiomegaly on the chest X-ray image.

## Results and discussions

This section demonstrates the results of each model and compare their efficiency based on distinct criteria for assessment. For this purpose, we use the test data which is not used in the training phase. First, we shall explain the classification report of each model and confusion matrix of our best model. Next, the ROC AUC scores of all the models are compared and then visualize some class-specific attention maps on the chest x-ray.

### Classification report

We obtain four measurements: accuracy, precision, recall, and F1-score using the test dataset. As demonstrated in Fig. 2b, the learning algorithms CXA_Res50, CXA_IncepRes2, CXA_Dense121, and CXA_Dense201 are used to obtain these measurements. The results of each model are shown in Table 4 for both classes: Non-Cardiomegaly and Cardiomegaly. We also calculate their average weighted by the number of true instances for each target.

**Table 4:** Classification report on the test datasets from four models.

Model	Class-Labels	Precision	Recall	F1-Score	Accuracy
CXA_Res50	Non-Cardio	0.88	0.80	0.84	
	Cardio	0.41	0.57	0.48	75%
	**Avg/total**	0.78	0.75	0.76	
CXA_IncepRes2	Non-Cardio	0.90	0.91	0.91	
	Cardio	0.61	0.59	0.60	84.75%
	**Avg/total**	0.85	**0.85**	0.85	
CXA_Dense201	Non-Cardio	0.89	0.86	0.88	
	Cardio	0.52	0.59	0.55	80.58%
	**Avg/total**	0.82	0.81	0.81	
CXA_Dense121	Non-Cardio	0.93	0.88	0.90	
	Cardio	0.59	0.73	0.66	**85%**
	**Avg/total**	**0.87**	**0.85**	**0.86**	

Significant values are in bold.

Precision, recall, f1-score of each model for both classes: non-cardiomegaly and cardiomegaly (cardio) along with the corresponding test accuracy.

We see that the precision of CXA_Dense121 is top among other designs by attaining 85 percent peak accuracy. The recall of the CXA_IncepRes2 model and the CXA_Dense121 model is similar by scoring 0.85. The average precision score of CXA_Dense121 is 0.87, highest in all. Thus, the model CXA_Dense121 produces highest F1-score by scoring 0.86 than other designs. We also perform several fundamental studies to comprehend the GWAP based AMM with GAP on the same baseline models. For a valid comparison, we apply these techniques to the same fundamental network architecture^35–37^. We exclude the last layers and replace them with a GWAP based AMM or GAP followed by a classification layer. Quantitative average results of each of these models are shown in Table 5.

**Table 5:** The performance comparison on the test dataset of the base-model-gap with the base-model-amm to the average precision, average recall, average f-1 score and auroc scores.

Model	Avg. Precision	Avg. Recall	Avg. F-1 Score	AUROC
ResNet50-GAP	**0.84**	0.70	0.73	0.86
CXA_Res50	0.78	**0.75**	**0.76**	**0.87**
InceptionRes2-GAP	0.84	0.81	0.82	**0.86**
CXA_IncepRes2	**0.85**	**0.85**	**0.85**	0.85
DenseNet201-GAP	**0.83**	0.66	0.69	0.84
CXA_Dense201	0.82	0.81	**0.81**	**0.86**
DenseNet121-GAP	0.84	0.68	0.71	0.85
CXA_Dense121	**0.87**	**0.85**	**0.86**	**0.89**

Significant values are in bold.

### Confusion matrix

**Figure 5:**
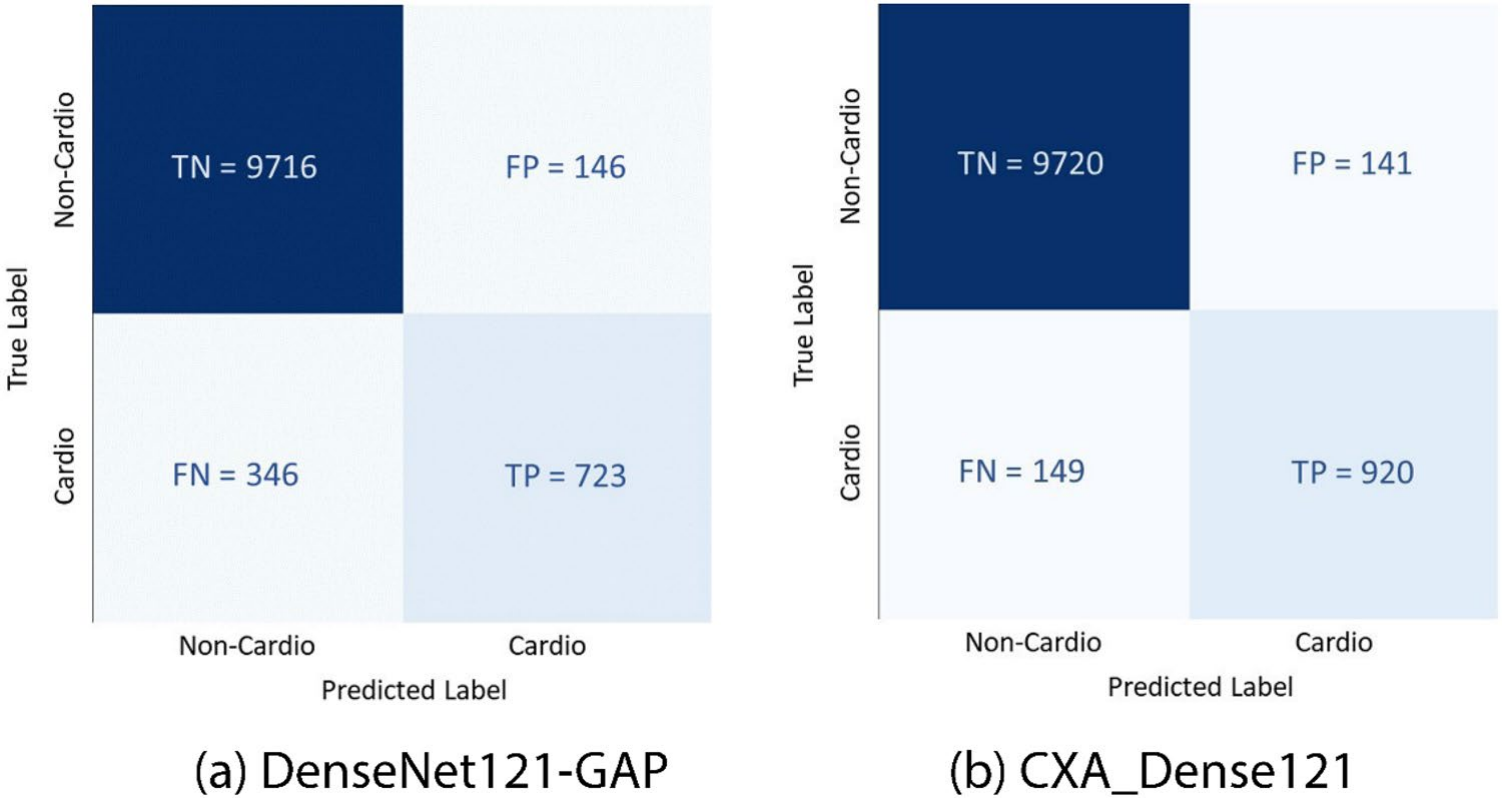
Comparison of confusion matrix of our (**a**) base model DenseNet121-GAP and (**b**) proposed best single model CXA_Dense121 under test dataset.

Figure 5 illustrates the confusion matrix obtained for our best model CXA_Dense121 (right) and its base model DenseNet121-GAP (left). It is found that CXA_Dense121 performs significantly better than its base model in terms of both true positive and true negative predictions. This indicates that the proposed CXA_Dense121 is a more robust classifier.

### ROC AUC curves

**Figure 6:**
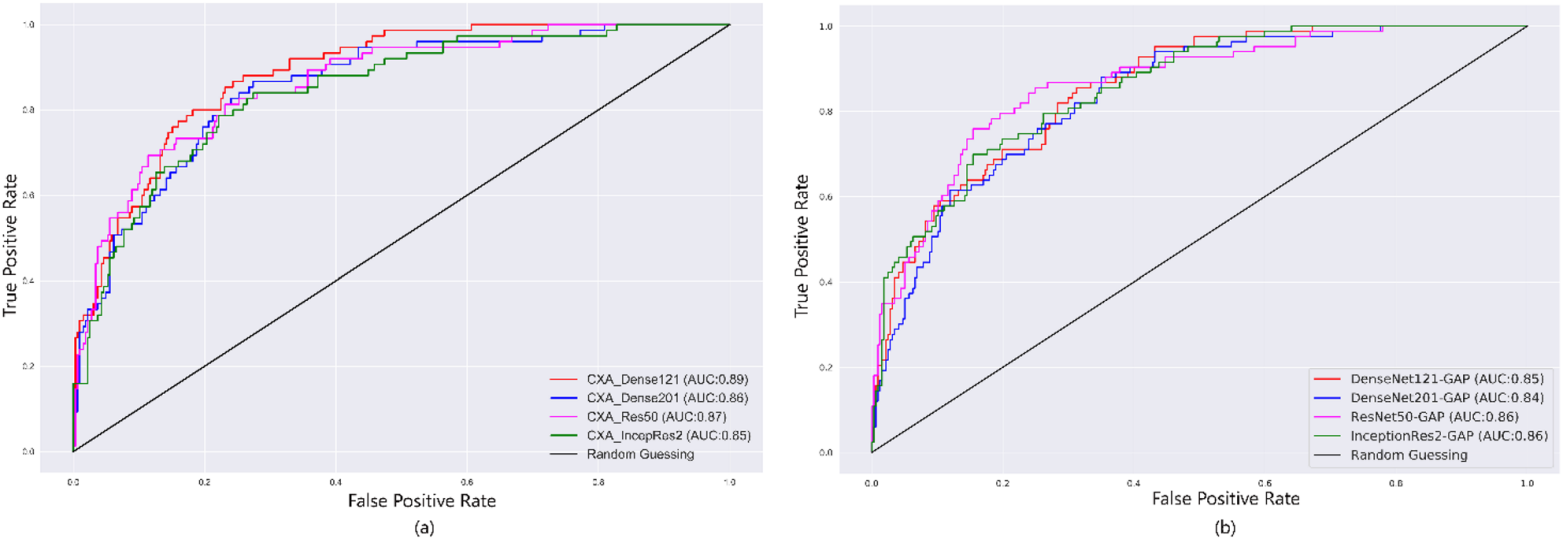
Comparison of ROC curves under Test dataset (**a**) for CXA_Dense121, CXA_Dense201, CXA_IncepRes2 and CXA_Res50; and (**b**) for DenseNet121-GAP, DenseNet201-GAP, ResNet50-GAP, and InceptionRes2-GAP.

The obtained AUROC scores of GWAP based AMM (left) and GAP (right) are shown in Fig. 6. The AUC scores of models CXA_Dense121, CXA_Dense201, CXA_IncepRes2 and CXA_Res50 are 0.89, 0.86, 0.85 and 0.87 respectively. Here the CXA_Dense121 outperforms other models followed by CXA_Res50. Among all the models, CXA_Dense121 appears as the strong classifier that produces a greater region under the ROC curve. The model operates well as a strong classifier that provides TPR close to unity while maintaining FPR close to zero.

### Visualization of class-specific attention map

In this part, we illustrate some heatmap visualization outcomes produced by the AMM. By leveraging the technique of the attention module, each baseline model yields very promising results. Along with properly classifying cardiomegaly class information at image-level, these models are also able to localize the most precise region on the chest x-ray to report cardiomegaly.

**Figure 7:**
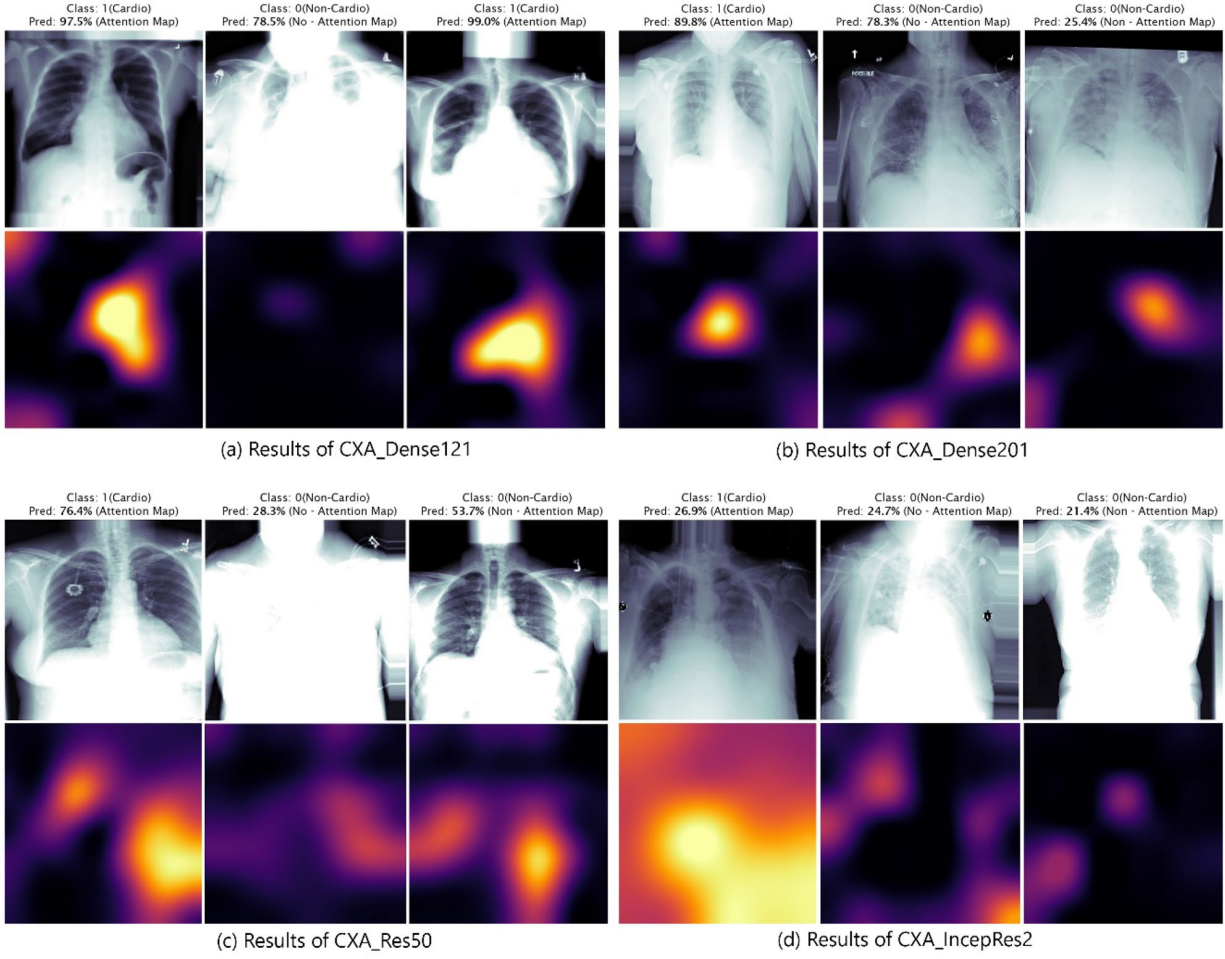
The results from attention map CXA_Dense121, CXA_Dense201, CXA_Res50 and CXA_IncepRes2 are shown in above (**a**), (**b**), (**c**) and (**d**) respectively. The ground truth of Class: 0 for Non-Cardiomegaly and 1 for Cardiomegaly are set. CXA_Dense121 model produces strong results compare to others for classifying cardiomegaly class information and localization on the most indicative area on the chest x-ray image.

A few images are selected randomly from the test dataset, as shown in Fig. 7, to demonstrate the visualization outcomes. It shows that CXA_Dense121 (Fig. 7a) strongly detects the cardiomegaly class information along with producing strong attention map on the precise location of the Chest-Xray images. We label the ground truth 1 and 0 for cardiomegaly and non-cardiomegaly respectively. The outcomes of other three models of CXA_Dense201, CXA_IncepRes2, and CXA_Res50 are also shown in Fig. 7b,c for better comparison. Each model shows promising outcomes to classify cardiomegaly class information and localization with the attention map. However, the proposed CXA_Dense121 model generates the most salient region maps on the chest x-ray while learning not to map for the non-cardiomegaly with highly classified confidences.

**Figure 8:**
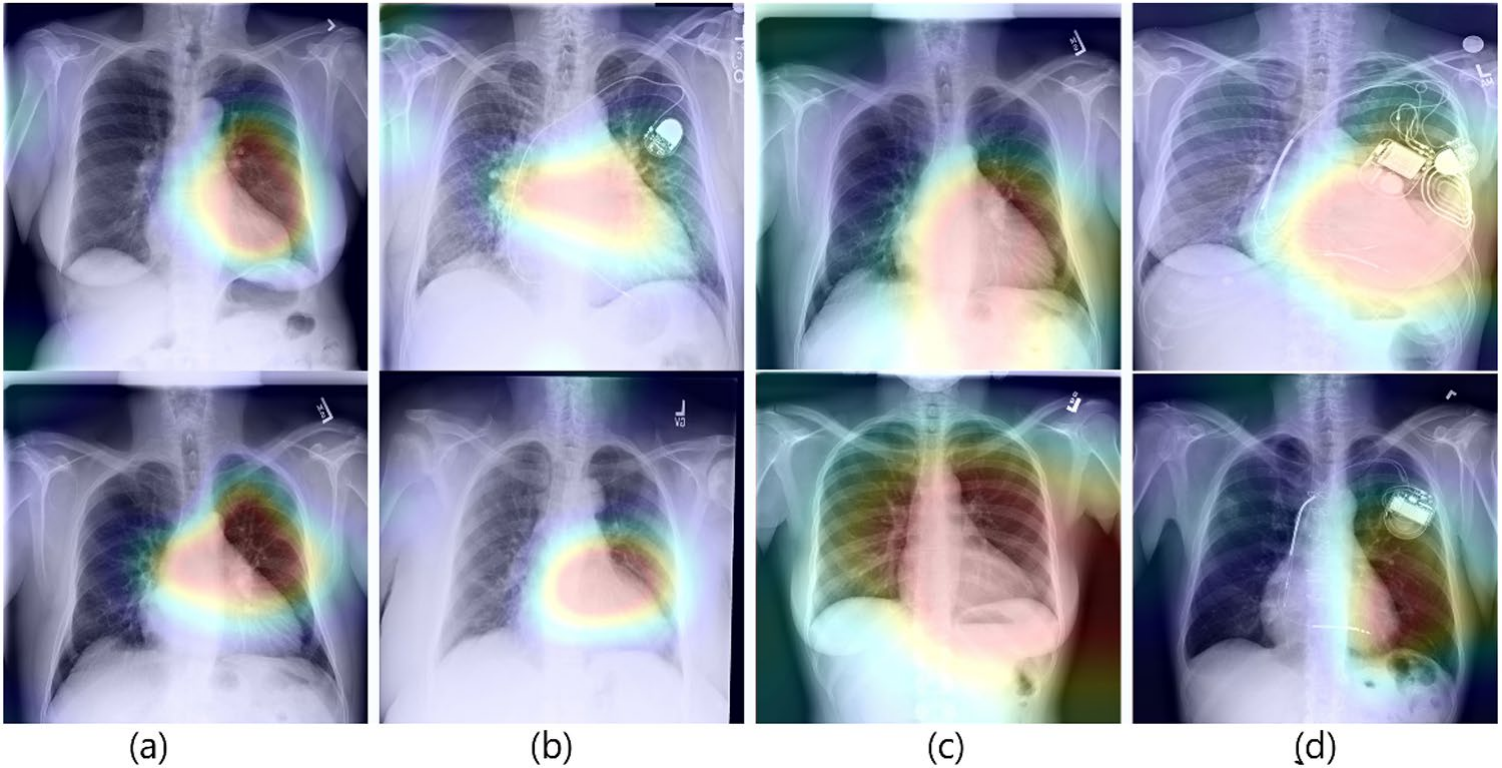
A visual illustration of superimposing the Attention Map on the corresponding cardiomegaly chest x-ray images with intensity factor 0.2. Outputs of CXA_Dense121 (**a**), CXA_Dense201 (**b**), CXA_IncepRes2 (**c**) and CXA_Res50 (**d**) are sequentially from left to right.

The localization outcomes using the proposed AMM can be better visualized by superimposing the attention maps on the corresponding x-ray images. In Fig. 8, we have shown some superimposed outcomes of the following models CXA_Dense121, CXA_Dense201, CXA_IncepRes2, and CXA_Res50. It is found in generally that the proposed AMM explicitly enables the ConvNet to have precise localization capabilities despite being trained on only image-level labels. The results also reveal that the CXA_Dense121 model substantially outperforms the other models considered in this work.

**Figure 9:**
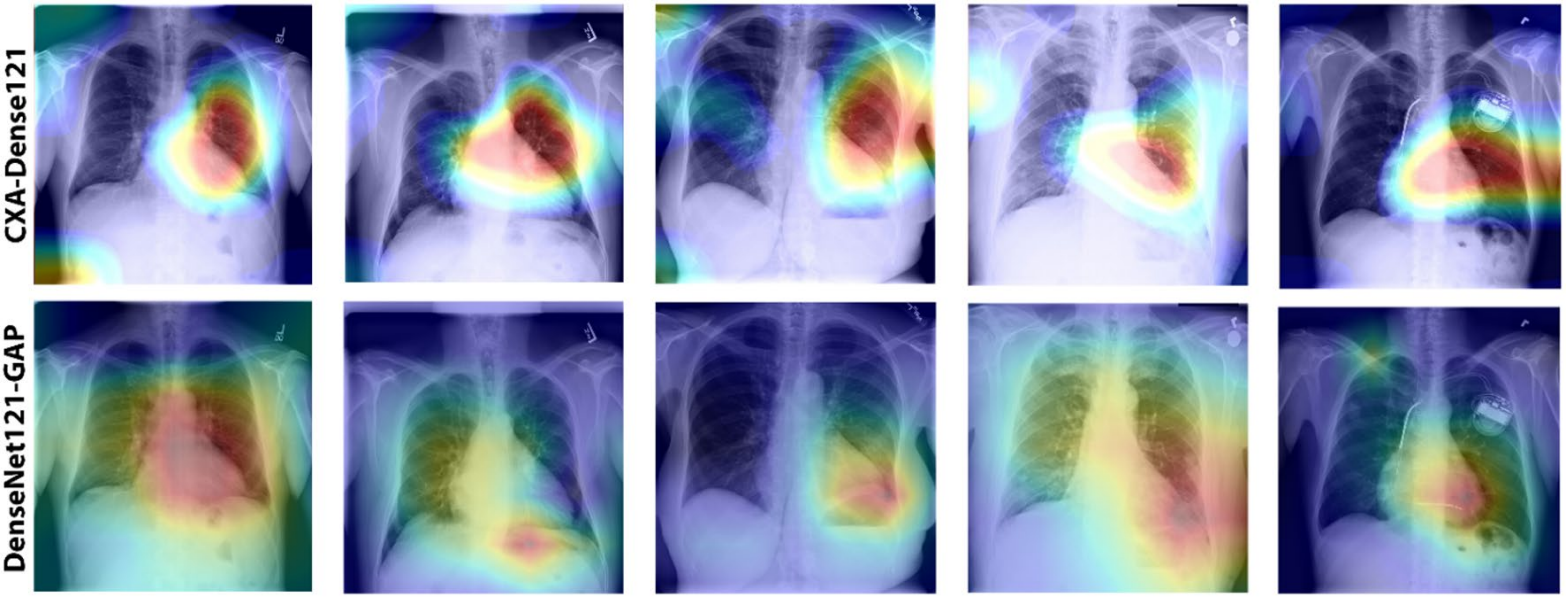
A visual demonstration with class-activation maps of our best model, DenseNet121 + AMM (or CXA_Dense121) and corresponding DenseNet121 + GAP model. The visual outcomes indicate that; the proposed attention mechanism method gives strong visual performance for interpreting the decision-making process.

To validate the effectiveness of our proposed method, we also visually demonstrate the class-activation maps of our best model (CXA_Dense121) in comparing with that of its base model (DenseNet121-GAP) on the cardiomegaly test images in Fig. 9. This indicates that the proposed method achieves better visual performance producing remarkable attention outcomes for the localization of the most indicative region on chest x-ray images, whereas the base model only could not produce clear visual outcomes. Such results are really promising since they are produced without any annotated bounding box.

However, there are several limitations to the proposed method. For example, out of the 2776 cardiomegaly cases in the ChestX-ray14 dataset, only 146 had manually annotated bounding boxes, which were not utilized in the proposed method. Additionally, multiple diseases often co-occur, and taking this into account could improve the accuracy of diagnosis. Finally, the disease labels in the dataset may be noisy, as they were extracted from radiological reports using natural language processing techniques, and this should be considered when designing the classification model.

## Conclusions

In this study, we examined the global weighted average pooling (GWAP) operation and developed the AMM network that enables the classification of cardiomegaly from the chest X-rays addressing with simultaneously image-level classification and pixel-level localization with only the image-level labels. To evaluate the empirical evidence of the proposed AMM, we leverage the advantages of some of the well-performed state-of-the-art ConvNet architectures (i.e. DenseNet-121, DenseNet-201, InceptionResNet_V2 and ResNet50). Finally, we develop the Cardio-XAttentionNet by incorporating these advanced ConvNet architectures as baseline models with the proposed attention mechanism (AMM). From our experiment, we achieved that Cardio-XAttentionNet based on DenseNet-121 (CXA_Dense121) appears as the well-generalized model and produced remarkable results in addressing cardiomegaly class information and localization simultaneously and substantially outperforms the other models. It achieves an overall precision, recall, F-1 measure and area under curve scores of 0.87, 0.85, 0.86 and 0.89 respectively for the classification of the cardiomegaly symptoms which is the state-of-the-art performance on this data set using a single model. Further, we demonstrated a comparative analysis on the effectiveness of the proposed mechanism (AMM) against the previous methods (GAP) for the classification of cardiomegaly as well.

As the visual proof supporting the results of classification is an inevitable part of clinical diagnosis, Cardio-XAttentionNet can provide high interpretation and deep insight. By showing the symptoms of cardiac insufficiency on the salient areas aligned with the visualization and high precision, Cardio-XAttentionNet can be a great AI tool to use in medical diagnosis for the radiologist and can be widely applied in clinical practice where thorough annotations are hardly available.

## Data Availability

All datasets used in this study are publicly available as indicated in “Data Set and Model Settings” section. Algorithmic implementations generated and analyzed during this study are available from the corresponding author on reasonable request.
